# Bioethanol production from potatoes in India: A SWOT analysis

**DOI:** 10.1016/j.heliyon.2024.e40677

**Published:** 2024-11-23

**Authors:** Dharmendra Kumar, Som Dutt, Arvind Kumar Jaiswal, Bandana Kaundal, Dinesh Kumar, Brajesh Singh

**Affiliations:** a ICAR-Central Potato Research Institute, Shimla, HP, India; b ICAR-Central Potato Research Institute, Regional Station, Jalandhar, Punjab, India; c ICAR-Central Potato Research Institute Regional Station, Modipuram, UP, India

**Keywords:** Bioethanol, Potatoes, India, SWOT analysis, Renewable energy

## Abstract

India is the third-largest energy consumer globally, heavily reliant on imported oil. Projections suggest India's energy consumption will double by 2050, posing challenges to energy security and leading to significant foreign currency outflows. The extensive use of fossil fuels increases carbon emissions, raising environmental and health concerns. In this context, bioethanol production from potatoes offers a promising solution. This paper presents a SWOT analysis of this potential.

Strengths include potatoes' widespread availability, high starch content, and compatibility with existing infrastructure. However, weaknesses such as seasonal cultivation, water-intensive farming, and competition with food demand require strategic solutions. Opportunities for bioethanol production from potatoes are numerous, including rural development, reduced fossil fuel reliance, and supportive governmental policies promoting renewable energy. Yet, threats like market fluctuations, technological limitations, and environmental issues related to land use and water consumption challenge the feasibility of this venture.

This SWOT analysis provides insights into the strengths, weaknesses, opportunities, and threats associated with bioethanol production from potatoes in India, highlighting the potential and challenges of this renewable energy pathway.

## 1 Introduction

As an agrarian nation, India has a significant agricultural output, including staple crops like potatoes. With increasing energy demands and a push towards greener fuels, the conversion of surplus agricultural products into biofuels presents an enticing opportunity [1,2]. Potatoes, being rich in starch, are a promising feedstock for bioethanol production [3,4].

The relentless rise in global energy consumption, fueled by swift industrial expansion, urban development, and population growth, has raised alarms about environmental deterioration, climate change, and the limited availability of fossil fuels [5]. Consequently, there's an escalating focus on shifting towards sustainable energy sources. Bioethanol, derived from biomass, has emerged as a promising substitute for fossil fuels in transportation, contributing to this transition [6].

India recognized as among the globe's swiftly advancing economies, confronts the dual imperative of fulfilling its escalating energy requisites while ameliorating ecological repercussions. The Indian administration acknowledges the significance of renewable energy and has established ambitious goals to elevate the portion of renewables within the energy amalgam. Amidst diverse renewable energy alternatives, bioethanol emerges as a particularly auspicious candidate owing to its capability to diminish greenhouse gas emissions, bolster energy fortification, and foster rural progression via biomass exploitation.

**Fig. 1:**
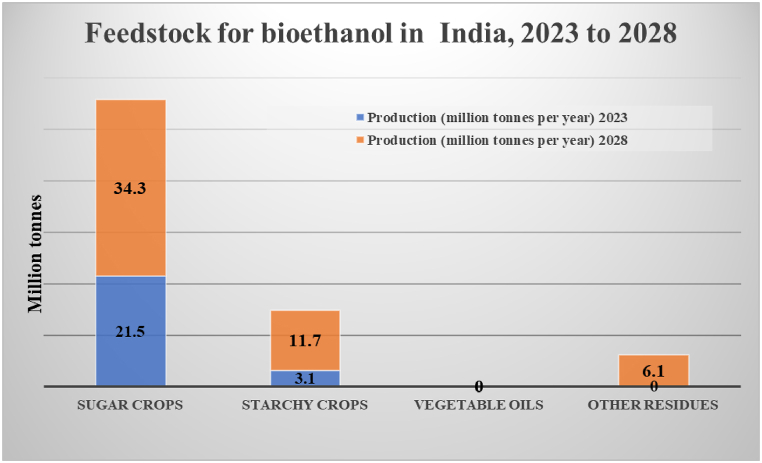
Feedstock for bioethanol in India, 2023 to 2028. Its provides data on the projected production of various feedstocks used for bioethanol production in India for the years 2023 and 2028. The feedstocks are sugarcane, starchy crops and other residues.

Fig. 1 provides data on the projected production of various feedstocks used for bioethanol production in India for the years 2023 and 2028 [7].

Table 1 provides a detailed overview of the roadmap or mandate for ethanol blending in different countries. Ethanol blending mandates vary significantly across different nations, reflecting diverse approaches to incorporating renewable fuels into transportation. In Brazil, the national biofuels policy initiated in 2015 mandates ethanol blending in gasoline at 18–27.5 %, with the current ratio set at 27 %, primarily used in flexible vehicles and two-wheelers. The USA, under the Clean Air Act, has the Environmental Protection Agency (EPA) setting annual volume requirements for the Renewable Fuel Standards (RFS), applicable mainly to conventional vehicles with higher blends like E30 or E85 designated for flex vehicles. The European Union's Renewable Energy Directive aims for a 10 % renewable fuel share in transportation by 2020, while China's 2017 legislation targets a 10 % ethanol blend nationwide. India's National Biofuels Policy of 2018 sets ambitious goals of 10 % ethanol blending in petrol by 2022, rising to 20 % by 2025, and a 5 % blend in diesel by 2030 [8] (see Table 2).

**Table 1:** Ethanol Blending Requirements and guidelines across different nations [9].

Country	Roadmap/Mandate for ethanol blends	Program	Implementation by	Vehicle Type
Brazil	Brazil's national policy maintains the requirement to blend ethanol at a rate of 18–27.5 % in gasoline, a mandate initiated in 2015. Presently, this blending ratio stands at 27 %.	National biofuels policy, 2017	Ministry of mines and energy (MME)	Primarily flexible, motorbikes and other two-wheeler engines utilize E27.
USA	The Clean Air Act mandates that the Environmental protection agency (EPA) establish the annual volume requirements for Renewable Fuel Standards (RFS). These volume requirements are updated by the EPA each year, taking into account fuel availability.	RFS program	EPA	Mainly conventional; Flex for exclusive use with E30 or E85
European Union (EU)	The EU targets to achieve a renewable source share, such as biofuels, comprising 10 % of the transportation fuel in every EU member country by 2020.	Renewable energy directive	European Commission	Flex and normal
China	In 2017, the Chinese government unveiled legislation advocating for the utilization of ethanol in fuel nationwide, aiming for a 10 % ethanol blending target across China.	Fuel quality standards	National Energy Administration	Primarily normal
India	The biofuels policy targets a 10 % ethanol blending in petrol by 2022, which will escalate to 20 % by 2025, and a 5 % blending in diesel by 2030.	National Biofuels Policy, 2018	Government of India	Flex and normal

**Table 2:** Annual global production of fuel ethanol (million gallons) [8].

Region	Bioethanol production (Mgal/y)					% of World Production
	2018	2019	2020	2021	2022	
USA	16,091	15,778	13,941	15,016	15,361	54 %
Brazil	8,060	8,860	8,100	7,320	7,400	26 %
EU	1,350	1,380	1,330	1,410	1,460	5 %
**India**	**450**	**500**	**520**	**870**	**1,230**	**4 %**
China	810	1,020	940	900	920	3 %
Canada	460	497	429	434	447	2 %
Thailand	390	430	390	350	370	1 %
Rest of World	699	645	620	680	722	3 %
Total	28,600	29,400	26,480	27,250	**28,220**	

Ethanol blending refers to the practice of mixing ethanol, typically derived from renewable sources like sugarcane, corn or others, with gasoline for use as a fuel in vehicles. This blending process is often mandated by governments as a strategy to reduce greenhouse gas emissions, promote energy security, and support the growth of the renewable energy sector.

India's current energy strategy is oriented towards achieving several key targets: generating 175 GW of renewable energy by 2022, reducing greenhouse gas emissions from the energy sector by 33%–35 %, and attaining a non-fossil fuel-based electricity mix of 40 % by 2030. Biofuels are recognized as crucial components in accomplishing these objectives. The primary focus concerning biofuels is to stimulate the local production of non-food feedstocks. The 2018 National Policy on Biofuels outlines specific targets, including achieving a 10 % ethanol blending in petrol by 2022, which is set to increase to 20 % by 2025, and a 5 % blending in diesel by 2030. Additionally, this policy broadens the spectrum of materials eligible for ethanol production, encompassing waste potatoes among other resources [10].

Since 2007, the United States has held the primary position in fuel ethanol production, with Brazil following closely behind. Collectively, these two countries contribute 84 % to the global ethanol production. The European Union, China, and Canada also play significant roles as leading fuel ethanol producers. Despite fluctuations in crude oil prices, both production and utilization of fuel ethanol are on the rise. Leading the world in fuel ethanol production is the United States, producing 60 billion litres primarily from corn, followed by Brazil with 33 billion litres sourced from sugarcane. While India holds the fourth position in fuel ethanol production, its output remains considerably smaller compared to the US and Brazil. India achieved a blending rate of 11.8 % in 2023 and aims to double it by 2024–25. It is noteworthy that India could benefit from Brazil's approach to mandatory blending and utilization of blended fuels in vehicles [8].

In this context, exploring the feasibility of bioethanol production from potatoes in India becomes pertinent. Potatoes are one of the most widely cultivated crops in India, with the country being the world's second-largest producer after China. The abundance of potatoes presents an opportunity to leverage this versatile crop as a feedstock for bioethanol production [11]. However, assessing the viability and potential of this venture requires a thorough analysis of its strengths, weaknesses, opportunities, and threats (SWOT).

The SWOT analysis will begin by examining the strengths of using potatoes as a feedstock for bioethanol production. Potatoes are rich in starch, which can be converted into fermentable sugars through enzymatic hydrolysis, making them an attractive feedstock for ethanol production. Furthermore, potatoes are widely cultivated across different regions of India, ensuring a consistent supply throughout the year. Additionally, the existing infrastructure for potato cultivation, storage, and processing can be repurposed for bioethanol production, reducing the need for substantial investment in new facilities.

However, along with strengths, it is essential to identify the weaknesses associated with bioethanol production from potatoes. One significant weakness is the seasonal nature of potato cultivation, which may result in fluctuations in feedstock availability and processing capacity utilization. Moreover, potatoes are water-intensive crops, and their cultivation can exacerbate water scarcity issues in certain regions. Furthermore, there is a potential conflict between using potatoes for bioethanol production and meeting food demand, especially in a country where food security is a priority.

Despite these weaknesses, there exist numerous opportunities for bioethanol production from potatoes in India. One notable opportunity is the potential for rural development through the establishment of bioethanol production units in potato-growing regions. This could create employment opportunities, enhance income levels, and contribute to the socio-economic development of rural communities. Additionally, bioethanol production from potatoes aligns with the government's objectives of promoting renewable energy and reducing dependency on fossil fuels. The Indian government has implemented various policies and incentives to support the renewable energy sector, providing a favorable regulatory environment for bioethanol production.

However, alongside opportunities, some threats need to be addressed to ensure the success of bioethanol production from potatoes in India. One significant threat is the volatility of the market for biofuels, influenced by factors such as fluctuating oil prices, government policies, and competition from other renewable energy sources. Technological constraints, such as the efficiency of ethanol conversion processes and the development of robust pretreatment methods for potato biomass, pose additional challenges. Moreover, environmental concerns regarding land use change, deforestation, and water consumption associated with potato cultivation need to be addressed to ensure the sustainability of bioethanol production from potatoes.

Bioethanol production from potatoes holds promise as a renewable energy option in India, leveraging the country's agricultural resources abundance and supporting its transition towards sustainable energy sources. However, a thorough SWOT analysis is essential to identify and address the various factors that may impact the viability and success of this venture. By examining the strengths, weaknesses, opportunities, and threats associated with bioethanol production from potatoes in India, this analysis aims to provide valuable insights for policymakers, investors, and stakeholders involved in the renewable energy sector.

This paper aims to conduct a comprehensive SWOT analysis of bioethanol production from potatoes in India. By systematically evaluating the internal and external factors affecting the viability of this endeavour, this analysis seeks to provide insights into the opportunities, challenges, and strategic considerations associated with utilizing potatoes as a feedstock for bioethanol production in India.

## 2 Bioethanol production

Bioethanol, a renewable fuel derived from biomass as feedstock, has gained significant attention as an alternative to fossil fuels due to its potential to mitigate greenhouse gas emissions and reduce dependency on non-renewable resources. In the pursuit of sustainable energy solutions, India, as one of the world's largest agricultural economies, has been exploring various avenues for bioethanol production. Among the diverse biomass resources available in India, potatoes emerge as a promising feedstock for bioethanol production due to their abundance and widespread cultivation across the country [[12], [13], [14]].

Bioethanol, also known as ethyl alcohol, is a renewable fuel produced through the fermentation of sugars derived from biomass feedstocks such as sugarcane, corn, wheat, and lignocellulosic materials. The conversion of biomass into bioethanol involves biochemical processes that typically utilize microorganisms such as yeast to ferment sugars into ethanol and other byproducts. Bioethanol can be used as a fuel additive or as a standalone fuel in vehicles, contributing to the reduction of greenhouse gas emissions and promoting energy security [15,16].

**Fig. 2:**
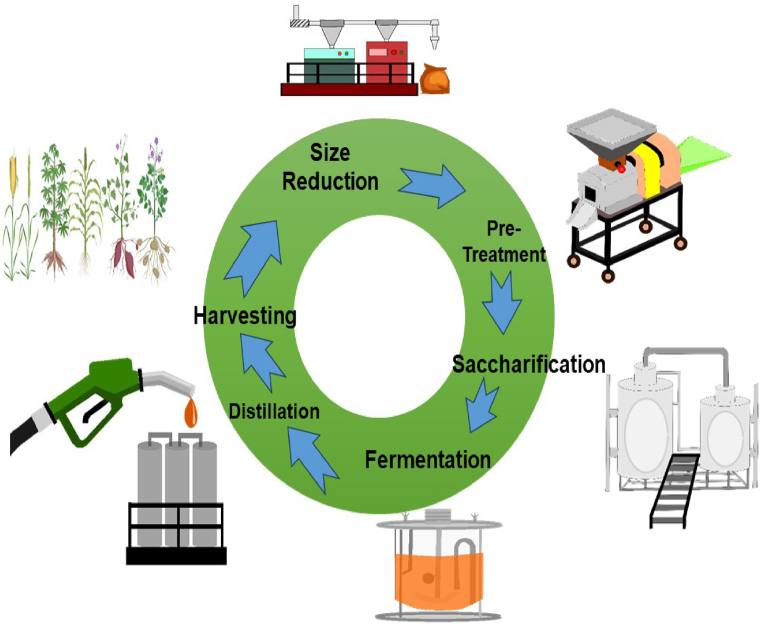
Bioethanol production process from potato. In the bioethanol production process from potatoes, starch is extracted through milling and grinding. Enzymes are then added to convert the starch into fermentable sugars, primarily glucose. Fermentation with yeast follows, producing ethanol and carbon dioxide. Distillation separates ethanol from the mixture, yielding a concentrated ethanol solution for further purification if needed.

Fig. 2 illustrates the bioethanol production process from potatoes, depicting the various steps involved in converting potatoes into ethanol, a renewable fuel source. The figure provides a visual representation of the process flow, highlighting key stages and components involved in bioethanol production.

The production of bioethanol typically involves the following steps [17,18].

Feedstock Preparation: Biomass feedstocks are harvested and processed to extract the fermentable sugars. For example, sugarcane and corn are crushed to extract their juice or starch, while cellulosic materials are pretreated to break down the cellulose into fermentable sugars.

Fermentation: The extracted sugars are fermented using yeast or bacteria in the presence of water to produce ethanol and carbon dioxide. Yeast species such as *Saccharomyces cerevisiae* are commonly used in ethanol production due to their efficiency in converting sugars to ethanol.

Distillation: The fermented mixture undergoes distillation to separate the ethanol from the water and other components. Distillation involves heating the mixture to vaporize the ethanol, which is then cooled and condensed back into a liquid form [19].

Dehydration: The ethanol obtained from distillation typically contains some water, which needs to be removed to increase the ethanol concentration. Dehydration processes such as molecular sieves or membrane technologies are used to remove the water and produce anhydrous (pure) ethanol [20].

Denaturing (optional): In some cases, ethanol is denatured by adding small amounts of substances such as gasoline or methanol to make it unsuitable for consumption as a beverage. Denatured ethanol is often used as a fuel additive or in industrial applications.

Byproduct Recovery: Throughout the production process, various byproducts such as carbon dioxide, stillage (residue from fermentation), and lignin (from cellulosic materials) are generated. These byproducts can be utilized for various purposes, including animal feed, biogas production, or as a source of additional revenue through further processing [[21], [22], [23], [24]].

The production of bioethanol offers several environmental benefits compared to fossil fuels, including reduced greenhouse gas emissions and lower dependence on finite fossil fuel resources. However, it also raises concerns related to land use, competition with food crops, and potential environmental impacts associated with intensive agriculture and biomass production.

Research and development efforts are ongoing to improve the efficiency and sustainability of bioethanol production, including the use of advanced feedstocks, optimization of fermentation processes, and integration with other renewable energy systems. Additionally, emerging technologies such as lignocellulosic ethanol and algae-based ethanol hold promise for further advancing the bioethanol industry.

## 3 Potato cultivation in India

Potatoes (*Solanum tuberosum*) are one of the most important staple crops in India, cultivated extensively across various regions and climatic conditions. India is among the top producers of potatoes globally, with a diverse range of varieties grown for both domestic consumption and export [25,26]. Potatoes are rich in starch, making them a suitable feedstock for bioethanol production through enzymatic hydrolysis and fermentation processes. Utilizing potatoes for bioethanol production not only offers a potential solution for surplus potato stocks but also creates value-added opportunities for farmers and stakeholders in the agricultural sector [4,22].

**Fig. 3:**
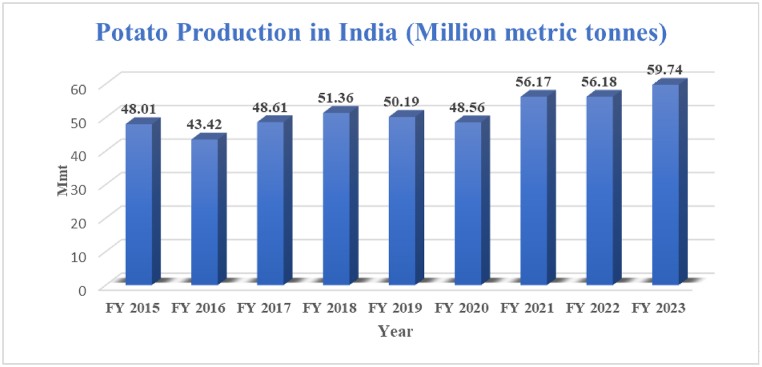
Trends in potato production in India. Its presents an analysis of the trends in potato production in India over the past decade, from 2010 to 2020. Potato production is a crucial component of India's agricultural sector, with significant implications for food security and economic stability.

**Fig. 4:**
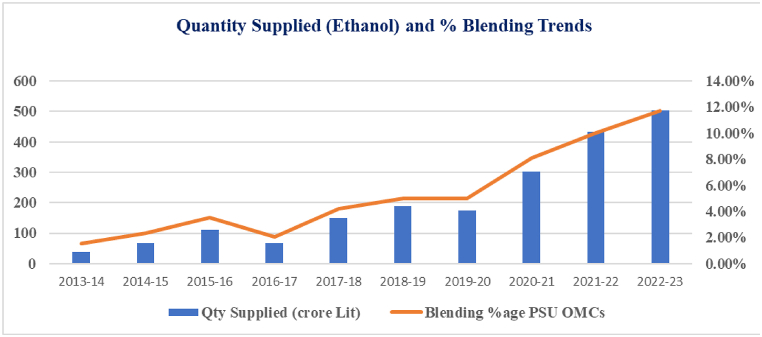
Illustrates the trends in the quantity of ethanol supplied and the percentage of blending by PSU OMCs over the years from 2013-14 to 2022–23. The data shows a consistent increase in ethanol supply from 38.0 crore Lit in 2013-14 to 502 crore Lit in 2022–23. Similarly, the blending percentage has steadily risen from 1.53 % in 2013-14 to 11.72 % in 2022–23, indicating.

Fig. 3 presents an analysis of the trends in potato production in India over the past decade, from 2010 to 2020. Potato production is a crucial component of India's agricultural sector, with significant implications for food security and economic stability [27]. Fig. 4 The trends in the quantity of ethanol supplied and the percentage of blending by PSU OMCs over the years from 2013-14 to 2022–23. The data shows a consistent increase in ethanol supply from 38.0 crore Lit in 2013-14 to 502 crore Lit in 2022–23. Similarly, the blending percentage has steadily risen from 1.53 % in 2013-14 to 11.72 % in 2022–23, indicating [28].

Potatoes are a valuable bioresource for bioethanol production due to their rich chemical composition. Comprising primarily water and carbohydrates, potatoes contain starch as the main carbohydrate source, accounting for about 15–20 % of their composition [26]. This starch can be enzymatically hydrolyzed into fermentable sugars such as glucose and maltose, essential for ethanol fermentation. Additionally, potatoes contain proteins, albeit in smaller quantities, providing essential nitrogen sources for microbial growth during fermentation [29,30]. The lipid content of potatoes, although low, can contribute to the overall energy yield in bioethanol production. Furthermore, potatoes harbor various vitamins and minerals, including vitamin C, vitamin B6, and potassium, which can act as nutrients for ethanol-producing microorganisms. Their fiber content, while modest, can influence the fermentation process and contribute to the overall nutritional value of the fermentation substrate [26,31,32]. Table 10 illustrates the biochemical composition of various Indian potato cultivars, focusing on moisture, starch, reducing sugars, and dry matter content. Among these, **Kufri Pukhraj** stands out with the highest moisture content (85.74 %) and a dry matter percentage of 14.25 %. In contrast, **Kufri Chipsona-3** and **Kufri Himsona** exhibit the highest dry matter percentages at 22.80 % and 22.99 %, respectively. **Kufri Surya** has the highest starch content at 86 %, while **Kufri Himsona** contains the lowest amount of reducing sugars at 130 mg/100g FW [33].

It can be concluded that potatoes represent a promising feedstock for bioethanol production, providing a varied chemical composition that can be effectively converted into bioethanol through pretreatment, enzymatic hydrolysis, and fermentation processes.

Moreover, in recent years, there have been efforts to increase potato cultivation and improve the productivity of the crop through various agricultural initiatives and technological advancements. Additionally, the government has implemented policies to support potato farmers and enhance the potato value chain in the country. Consequently, the availability of potatoes as a potential bioethanol feedstock could be sustainable.

## 4 SWOT analysis of bioethanol production from potatoes in India

### 4.1 Strengths

#### 4.1.1 High starch content

Potatoes are composed of approximately 15–20 % starch, making them a suitable raw material for ethanol fermentation. Bioethanol production from potatoes involves utilizing various components present in their nutritional composition, particularly focusing on starch and sugars. In Table 3 detailing the nutritional value of 100 g of fresh potatoes, starch stands out as a significant component at 15.9 g. Starch serves as the primary source of fermentable sugars for bioethanol production. Additionally, the presence of sugars like glucose, fructose, and sucrose, totaling 0.62 g, further contributes to the fermentable substrate.

**Table 3:** Nutritional value of potato (100 g fresh) [34].

Components	Amount
Water	78.6 g
Energy	79 kcal
Carbohydrate	18.1 g
Starch	15.9 g
Fiber, Total dietary	1.3 g
Total lipid (fat)	0.08 g
Ash	1.13 g
Total sugars	0.62 g
Sucrose	0.13 g
Glucose	0.25 g
Fructose	0.23 g
Potassium	417 mg
Vitamin C	5.7 mg

These sugars can be enzymatically hydrolyzed from the starch matrix during saccharification, facilitating their conversion into ethanol through fermentation. Potassium, an essential mineral found abundantly in potatoes at 417 mg, can act as a nutrient source for yeast during fermentation, supporting ethanol production (USDA, 2019). Moreover, the relatively low lipid and protein content of potatoes, along with their ash content, ensure a more efficient fermentation process with fewer undesirable byproducts.

#### 4.1.2 Abundant production

India is the second-largest producer of potatoes globally, implying a vast feedstock source. The potato cultivation landscape in India not only serves as a cornerstone of the nation's agricultural sector but also holds immense potential for bioethanol production. With vast expanses of land dedicated to potato farming across states like Uttar Pradesh, West Bengal, and Punjab, India boasts a substantial area under cultivation. This extensive cultivation area translates into a significant annual production of potatoes, positioning India as one of the world's top producers of this versatile tuber [35,36].

Moreover, the average yield per hectare of potatoes in India reflects steady improvement over time, driven by advancements in agricultural techniques and improved varieties. Given the high starch content of potatoes, they serve as an ideal feedstock for bioethanol production, offering a renewable and environmentally sustainable alternative to traditional fossil fuels. Government initiatives and agricultural policies further incentivize the utilization of potatoes for bioethanol production, fostering innovation and investment in this burgeoning sector.

India’s projected potato production capacity is expected to grow substantially by 2030. In 2022, potato production reached 53.5 million tonnes, showing an annual growth rate of around 4 %. By 2030, forecasts suggest production could reach between 65 and 70 million tonnes, depending on technological advancements and improved farming practices. This increase in production is being driven by rising demand, government support, and innovations in potato farming. India’s focus on boosting agricultural efficiency and productivity is likely to further enhance its potato output in the coming years.

As a result, the integration of potato cultivation with bioethanol production presents a promising avenue for enhancing both agricultural productivity and energy security in India, while also contributing to global sustainability efforts.

Over the decades, there has been a notable increase in both the potato cultivation area and production in India. The potato cultivation area has steadily risen from 0.24 million hectares in 1950-51 to 2.20 million hectares in 2020–21, indicating the expansion of potato farming across the country. Similarly, potato production has seen a significant increase, from 1.66 million metric tons in 1950-51 to 56.17 million metric tons in 2020–21 [27]. This substantial production growth reflects advancements in agricultural practices, technology, and infrastructure supporting potato cultivation in India.

Moreover, the yield per hectare of potatoes has also shown improvement over the years, increasing from 69.17 quintals/hectare in 1950-51 to 254.98 quintals/hectare in 2020–21. This increase in yield can be attributed to various factors such as the adoption of high-yielding varieties, improved irrigation techniques, and better management practices by farmers (Table 4) [27].

**Table 4:** Potato cultivation area, production, and yield in India [27].

Year	Potato area, production and yield in India		
	Area (mha)	Production (mt)	Yield (q/ha)
1950–51	0.24	1.66	69.17
1960–61	0.38	2.72	72.51
1970–71	0.48	4.81	99.78
1980–81	0.73	9.67	132.58
1990–91	0.94	15.21	162.54
2000–01	1.22	22.49	184.04
2010–11	1.86	42.34	227.24
2020–21	2.20	56.17	254.98

The market size of potatoes in India is projected to experience significant growth over the next five years, driven by several key factors. As of 2023, the Indian potato market was valued at approximately $40 billion and is expected to grow at a CAGR of 4–5% from 2024 to 2029. This growth can be attributed to the increasing demand for potatoes in both domestic and export markets, driven by their widespread use as a staple food and in processed forms, such as chips, fries, and other snacks. Additionally, advancements in agricultural practices and government initiatives to improve yield and production efficiency are likely to contribute to the market expansion. The growth in the potato processing industry, coupled with rising consumption patterns, particularly in urban areas, is also expected to play a crucial role in driving the market size. With these factors in play, the Indian potato market is poised to reach an estimated value of over $50 billion by 2029, reflecting the increasing importance of potatoes in the country's agricultural and food sectors.

Overall, the data highlights the remarkable progress and importance of potato cultivation in India, contributing significantly to the country's agricultural output and food security.

#### 4.1.3 Local availability

Localized production and processing can help reduce transportation costs and carbon footprint. Mostly cold storage is available in the potato growing region. The primary potato producers in Uttar Pradesh and West Bengal collectively account for approximately 55–56 % of the total domestic cold storage capacity, as indicated in Table 5.

**Table 5:** State-wise distribution of cold storage in India [37].

State	No. of Cold Storage	Total Capacity (MT)	Percent Capacity
Uttar Pradesh	2472	15.04	38.16
West Bengal	515	5.95	15.10
Gujarat	1014	3.97	10.07
Punjab	761	2.59	6.57
Andhra Pradesh & Telangana	467	1.89	4.79
Bihar	313	1.48	3.75
Madhya Pradesh	315	1.36	3.45
Maharashtra	656	1.18	3.00
Haryana	382	0.87	2.20
Karnataka	250	0.84	2.13
Rajasthan	191	0.65	1.65
Others	1317	3.59	9.10
**Total**	**8653**	**39.41**	**100.00**

Presently, the private sector owns 95 % of the cold storage facilities, while cooperatives and public sector undertakings own 3 % and 2 % respectively. There is a necessity to establish a system for real-time monitoring of stock levels to enhance the efficient utilization of storage capacity. As of November 20, 2023, the cold storage infrastructure in India exhibits a diverse distribution across different states, crucial for preserving perishable goods and reducing post-harvest losses. Uttar Pradesh stands out as the leader in this regard, boasting 2472 cold storage units, contributing 15.04 % to the total storage capacity, and making up 38.16 % of the national capacity. Following Uttar Pradesh is West Bengal, with 515 units, representing 5.95 % of the total capacity and 15.10 % of the national capacity. Gujarat, Punjab, and Andhra Pradesh & Telangana also play significant roles, each hosting over 400 cold storage units. Bihar, Madhya Pradesh, and Maharashtra, although with fewer units, still contribute substantially to the overall storage capacity [37].

The collective efforts of these states, along with others not specifically mentioned, account for the comprehensive cold storage network in India, crucial for ensuring food security and supporting the agricultural supply chain nationwide.

#### 4.1.4 Potential feedstocks for bioethanol production

The ethanol production potential of various crops listed in Table 6 underscores the versatility of plant-based materials as ethanol feedstocks. Virtually any plant material can serve as a feedstock for ethanol production, as they contain fermentable sugars in their stalks or grains, suitable for biochemical conversion into ethanol. Additionally, plant material can undergo thermochemical conversion processes to yield ethanol. The selection of a feedstock depends on multiple factors, such as the ease of cultivation of a particular crop, geographical advantages, and other potential uses like food and feed. Therefore, Table 6 offers valuable insights into the ethanol production potential of different crops, aiding in the decision-making process for selecting suitable feedstocks based on various considerations [35].

**Table 6:** Potential crops for biofuel production in India [10].

Crop	Ethanol yield (litres/t)	Crop/stalk yield (t/ha)	Ethanol production (litres/ha)
Sugarcane	70	80	5600
Sugar beet	100	50.6	5060
Sweet potato	125	18.8	2350
**Potato**	**110**	**20.9**	**2304**
Maise	360	5.9	2133
Cassava	180	11.3	2034
Rice	430	4.7	2012
Wheat	340	3.4	1165
Sweet sorghum	26.3	38.5	1013
Barley	250	3.0	738

#### 4.1.5 Enhanced rural employment

Bioethanol production can lead to the creation of job opportunities in rural areas. The production of bioethanol from potatoes presents a promising avenue for enhancing rural employment in India. From being known as providers of food to becoming suppliers of energy. By 2025, with a 20 % blending rate, the demand for ethanol is projected to reach 1016 crore litres. Consequently, the value of the ethanol industry is set to skyrocket by more than 500 %, soaring from approximately 9,000 crore to over 50,000 crore. A long-term agreement has been established to facilitate the creation of 431 crore litres per annum of dedicated ethanol production capacity. Additionally, an estimated surplus of 165 LMT of grain annually from 2025 will be diverted towards ethanol production, resulting in an approximate payment of 42,000 crore to farmers. The introduction of new vehicles capable of running on E20 fuel starting from 2023, followed by flex fuel vehicles in 2024, is expected to attract fresh investments and generate employment opportunities [28].

As a renewable and eco-friendly alternative to fossil fuels, bioethanol production not only contributes to reducing carbon emissions but also fosters economic growth, particularly in rural areas. With India being one of the largest producers of potatoes globally, leveraging this abundant agricultural resource for bioethanol production can unlock significant job opportunities in rural communities. From cultivating and harvesting potatoes to processing them into bioethanol, each stage of the production process offers employment prospects for local farmers and workers. Furthermore, establishing bioethanol plants in rural regions creates ancillary job opportunities in transportation, logistics, and other support services. By tapping into the potential of bioethanol production from potatoes, India can not only address energy security concerns but also bolster rural economies by generating sustainable employment opportunities, thereby contributing to inclusive growth and development [38].

#### 4.1.6 Post-harvest losses

Effective post-harvest management is crucial for extending the shelf life and enhancing the utility of potatoes. Studies have consistently shown that potatoes cultivated in tropical and sub-tropical climates incur significant losses, ranging from 30 % to 50 %, due to inadequate handling and storage practices. These losses can be categorized as qualitative or quantitative. Qualitative losses, stemming from physiological or pathological factors, substantially reduce the market value of potatoes. Harvesting and post-harvest activities, including handling, storage, and transportation, are integral to crop production, but each stage is susceptible to food losses, ultimately diminishing the available food quantity for consumption, known as quantitative losses [39,40].

Potatoes harvested from field storage can experience losses of up to 16 % within 20 days at the wholesaler, retailer, or household levels, primarily due to transpiration and rotting processes. The storage conditions at the time of harvest significantly influence these losses throughout the marketing chain. Potatoes stored at lower temperatures demonstrate reduced losses, ranging from 9 % to 14 % [41]. Certainly, these potato wastes hold the potential to be effectively utilized in ethanol production, contributing to sustainable resource management and the development of renewable energy sources.

### 4.2 Weaknesses

#### 4.2.1 Less yield compared to sugarcane

Compared to other sources like sugarcane and corn, potatoes might have slightly lower ethanol yields. In Table 7, which outlines feedstock cost and ethanol yield, each feedstock's cost per metric ton (MT), the quantity of ethanol produced per MT of feedstock, and the ex-mill ethanol price in Rs./litre are detailed. Among the various feedstocks listed, potato stands out for its unique characteristics in this context [9].

Potato, priced at Rs. 10 per kilogram or Rs. 10,000 per metric ton, exhibits a comparatively higher feedstock cost. However, its ethanol yield is noteworthy, with 110 L of ethanol produced per metric ton of potato. This substantial ethanol yield indicates the potential efficiency of potato as a feedstock for ethanol production. Moreover, the ex-mill ethanol price for potato-derived ethanol is significantly higher at Rs. 95 per litre compared to other feedstocks listed in Table 7.

**Table 7:** Feedstock cost and ethanol yield [9].

Feedstock	Cost/MT of the feedstock (Rs)	Quantity of ethanol per MT of feedstock	Ex-mill Ethanol Price (Rs/lt)
**Sugarcane juice**	2850 (10 % sugar recovery)	70 L	62.65
**B Molasses**	13,500	300 L	57.61
**C Molasses**	7123	225 L	45.69
**Broken Rice**	16,000	400 L	51.55
**Rice available with FCI**	20,000	450 lire	56.87
**Maize**	15,000	380 L	51.55
**Potato**	10,000 (Rs. 10/Kg)	110 L	95

This higher price point reflects the market value attributed to ethanol derived from potatoes, possibly due to factors such as processing costs, market demand, and the overall supply-demand dynamics in the ethanol industry (Table 8) [42].

**Table 8:** Price comparison of bioethanol in India [42].

Country	Prices per litre in USD
USA	0.694 (INR: 57.669)
Brazil	0.717 (INR: 57.669)
France	0.980 (INR: 57.669)
Thailand	0.998 (INR: 57.669)
Sweden	1.341 (INR: 57.669)
Switzerland	1.784 (INR: 57.669)
Spain	2.104 (INR: 57.669)
India∗	0.865 (INR 62.65)
Average	1.23 (INR: 102.30)

#### 4.2.2 Competition with food supply

Utilizing potatoes for bioethanol can compete with food supply, potentially raising food prices. The utilization of potatoes for bioethanol production presents a significant concern regarding competition with the food supply, which could potentially lead to increased food prices. Potatoes are a staple food crop consumed by millions worldwide, and diverting a portion of the potato harvest towards bioethanol production reduces the available supply for food consumption. This reduction in the food supply can create competition between the biofuel industry and the food market, ultimately driving up food prices as demand exceeds supply [43].

Such competition for resources raises ethical and socioeconomic concerns, particularly in regions where potatoes are a dietary staple or where food insecurity is prevalent. Therefore, careful consideration must be given to the implications of using potatoes for bioethanol production, balancing the potential benefits of renewable energy production with the need to ensure food security and affordability for all segments of the population. Strategies such as promoting the use of non-food feedstocks or implementing policies to mitigate the impact on food prices may be necessary to address these challenges effectively [5,44].

#### 4.2.3 Seasonal variability

Potato cultivation is subject to seasonal variations, leading to fluctuations in feedstock availability and production volumes for bioethanol. Potato cultivation is susceptible to seasonal variability, which poses challenges for bioethanol production due to fluctuations in feedstock availability and production volumes. The growth and yield of potatoes are influenced by various seasonal factors such as temperature, rainfall, and soil conditions. These factors can vary significantly from one growing season to another, leading to unpredictable yields and availability of potatoes for bioethanol production [45].

Seasonal variations in potato cultivation can also impact the overall supply chain, including transportation, storage, and processing, further complicating the production of bioethanol from this feedstock [46].

As a result, the biofuel industry relying on potatoes as a feedstock must contend with the inherent uncertainties associated with seasonal variability, requiring flexible production strategies and contingency plans to manage fluctuations in feedstock availability and production volumes effectively. Additionally, efforts to diversify feedstock sources or invest in technologies that mitigate the impact of seasonal variability on bioethanol production may help address these challenges in the long term [[46], [47], [48]].

#### 4.2.4 High cost of bioethanol production

The processing of potatoes into bioethanol involves significant costs, including enzymatic hydrolysis, fermentation, and distillation, which may affect the overall economic feasibility.

#### 4.2.5 Initial capital costs

Establishing new bioethanol plants requires significant capital investment. Establishing new bioethanol plants utilizing potato as a feedstock necessitates a substantial initial capital investment due to various factors involved in plant setup and operation [49]. The capital costs associated with such ventures primarily include expenses related to land acquisition, construction of the facility, procurement of specialized equipment and machinery for potato processing and ethanol production, installation of fermentation and distillation units, and implementation of quality control measures to ensure efficient and consistent ethanol production. Additionally, significant investment is required for utilities infrastructure, including water supply, energy sources, and waste management systems, to support the plant's operations sustainably [50].

Moreover, compliance with regulatory standards and obtaining necessary permits further adds to the initial capital outlay. Despite the significant upfront investment, establishing bioethanol plants from potatoes holds long-term potential for generating substantial returns on investment, given the favorable ethanol yield and market value associated with potato-derived ethanol, coupled with the increasing demand for renewable and sustainable fuel sources worldwide. Therefore, while the initial capital costs may be daunting, prudent investment in establishing bioethanol plants from potatoes can yield promising economic and environmental benefits in the long run [51,52].

### 4.3 Opportunities

#### 4.3.1 Research and development

Investment in R&D can lead to breakthroughs in enhancing conversion efficiency. Continued research and development in biotechnology and process optimization can enhance the efficiency and cost-effectiveness of bioethanol production from potatoes [53]. Investment in research and development presents significant opportunities for enhancing the conversion efficiency of bioethanol production from potatoes in India. Through targeted R&D initiatives, there exists the potential to achieve breakthroughs in various aspects of the ethanol production process, leading to improved efficiency, cost-effectiveness, and sustainability of potato-based ethanol production. Research efforts can focus on several key areas to unlock these opportunities [54].

Firstly, R&D can be directed towards developing advanced processing technologies specifically tailored for potato-based ethanol production. This includes innovations in enzymatic hydrolysis, fermentation techniques, and distillation processes aimed at maximizing ethanol yield from potato feedstock while minimizing energy consumption and production costs [55].

Secondly, genetic modification and breeding programs can be explored to enhance the starch content and fermentability of potato varieties used for ethanol production. This could involve genetic engineering techniques to optimize starch biosynthesis pathways or selecting and breeding potato cultivars with traits conducive to ethanol production, such as high starch content, rapid growth, and resistance to pests and diseases [56].

Furthermore, R&D investments can facilitate the development of integrated biorefinery concepts where various by-products and waste streams generated during potato processing, such as potato peels and pomace, are valorized to produce additional high-value products alongside ethanol, thereby improving overall process economics and resource utilization [35].

Additionally, research efforts can focus on improving sustainability aspects of potato-based ethanol production, including reducing water and energy consumption, minimizing waste generation, and exploring alternative sources of energy for powering ethanol plants, such as renewable energy sources like solar or biomass [57]. The production of bioethanol from potato biomass has gained significant attention due to the crop's high starch content and its potential as a sustainable energy source. Recent advancements in genomics and genome editing, particularly CRISPR-Cas9 technology, have enabled the development of genetically modified (GMO) potato strains that enhance starch yield and improve fermentability. These modifications can lead to more efficient saccharification processes, where starch is enzymatically broken down into fermentable sugars. Advanced fermentor technologies, such as continuous stirred-tank fermentors (CSTFs) and membrane bioreactors, optimize the fermentation environment by maintaining optimal temperature, pH, and nutrient levels, which are critical for microbial activity. The utilization of engineered microbial strains, including high-yield Saccharomyces cerevisiae and recombinant bacteria, can significantly improve fermentation efficiency by enhancing sugar uptake and ethanol tolerance. Furthermore, pre-treatment methods, such as enzymatic hydrolysis and steam explosion, are crucial in breaking down the potato cell wall and releasing fermentable sugars. These pre-treatment techniques can be optimized through genomic insights to develop more robust microbial strains capable of thriving in harsh conditions, thereby improving overall bioethanol yield. Integrating these innovative approaches enhances the economic feasibility of bioethanol production from potato, positioning it as a viable alternative to fossil fuels and contributing to a more sustainable energy future [58,59].

Overall, investment in R&D holds immense potential for driving innovation and efficiency improvements in bioethanol production from potato in India, paving the way for a more sustainable and economically viable ethanol industry while contributing to the nation's energy security and environmental goals.

#### 4.3.2 Blending mandate

The Indian government's initiative to blend ethanol with petrol has the potential to stimulate growth in the bioethanol industry. The commercial production and marketing of ethanol-blended gasoline began in January 2003. Initially, during the initial phase of the Ethanol Blending Programme (EBP), a mandate for blending 5 % ethanol in gasoline was introduced in nine states and four union territories [60].

Later, in August 2005, the government facilitated an agreement between the sugar industry and petroleum companies to streamline ethanol procurement. Following a significant increase in sugarcane/sugar production in 2006–07, the Government of India announced the second phase of the EBP in September 2006, requiring 5 % ethanol blending with petrol in 20 states and eight union territories, subject to commercial feasibility [28].

However, despite policy support and surplus sugarcane and sugar production, the targeted 5 % blending under the EBP could not be achieved. The actual quantity of ethanol supplied fell significantly short of the tendered quantity (Table 9), mainly due to supply-side constraints such as limited distillation capacity and molasses availability. It is evident that while there is progress in fuel ethanol demand, current production levels are inadequate to meet the blending targets of 10 % by 2022 and 20 % by 2025.

**Table 9:** Outlines the anticipated petrol demand and corresponding ethanol requirements in India [60].

Year	Petrol demand (MML)	Ethanol blending requirements (MML)			
		5 %	10 %	15 %	20 %
2019–20	37140	1857	3714	5571	7418
2024–25	49482	2227	4453	6680	6680
2029–30	60203	2709	5418	8127	10836

**Table 10:** Biochemical composition of indian potato cultivars [32].

Potato Variety	Moisture (%)	Starch (%) (g/100 g)	Reducing Sugars (mg/100 g FW)	Dry matter (%)
Kufri Anand	84.69	62	367	15.30
Kufri Arun	79.75	77	391	20.24
Kufri Badshah	82.71	78	446	17.28
Kufri Bahar	78.86	60	210	21.13
Kufri Chandramukhi	80.09	68	252	19.90
Kufri Chipsona-1	81.21	65	286	18.78
Kufri Chipsona-2	80.94	63	384	19.05
Kufri Chipsona-3	77.19	67	254	22.80
Kufri Frysona	78.92	57	275	21.07
Kufri Girdhari	81.92	82	347	18.07
Kufri Himalini	82.72	59	370	17.27
Kufri Himsona	77	75	130	22.99
Kufri Jyoti	82.04	70	252	17.95
Kufri Khyati	85.55	66	347	14.44
Kufri Lalima	82.34	79	305	17.65
Kufri Pukhraj	85.74	59	388	14.25
Kufri Surya	82.22	86	225	17.77

Table 9 provides official estimates of the ethanol requirement for blending with petrol at levels of 5 %, 10 %, 15 %, and 20 %, based on the demand for petroleum products.

#### 4.3.3 Waste utilization

Utilizing potato waste and peels for ethanol production presents a significant opportunity to mitigate waste generation and enhance the sustainability of the potato processing industry in India. Potato waste, including peels, stems, and culls, constitutes a substantial portion of the by-products generated during potato processing. Instead of being discarded or left to decompose, these waste materials can be redirected for ethanol production through various technological processes [35].

In India, where potato cultivation is widespread and forms a crucial part of the agricultural sector, the volume of potato waste generated is substantial. According to estimates, approximately 20–30 % of the total potato production in India ends up as waste, amounting to millions of metric tons annually. This waste poses environmental challenges, including soil and water pollution, methane emissions from decomposition, and the loss of valuable organic matter [61].

Redirecting potato waste and peels for ethanol production not only addresses the issue of waste management but also contributes to the production of renewable fuel and reduces dependency on fossil fuels. Ethanol production from potato waste involves biochemical processes such as enzymatic hydrolysis and fermentation, where starch and other carbohydrates present in the waste material are converted into ethanol.

#### 4.3.4 Export potential

If efficiently produced, India could become a bioethanol exporter, tapping into the international biofuel market. The efficient production of bioethanol in India opens up significant opportunities for the country to become a bioethanol exporter, tapping into the rapidly growing international biofuel market. With the increasing global focus on reducing greenhouse gas emissions and transitioning towards renewable energy sources, there is a rising demand for biofuels, including ethanol, across various sectors such as transportation, energy generation, and industrial applications [60,62].

India, with its abundant agricultural resources and favorable climatic conditions, possesses a strong competitive advantage in bioethanol production. The country's vast agricultural lands can be leveraged to cultivate feedstocks like sugarcane, maize, rice, and even potatoes, which can be efficiently converted into ethanol through advanced processing technologies. Moreover, India has a well-established sugar industry that produces significant quantities of molasses, a key feedstock for ethanol production [63].

By optimizing production processes, enhancing efficiency, and leveraging economies of scale, India can produce bioethanol at competitive prices, making it an attractive supplier in the global biofuel market. Additionally, India's strategic geographical location provides easy access to key international markets in Asia, Europe, and beyond, facilitating export logistics and trade partnerships [64]. Furthermore, India's participation in the international biofuel market can bolster diplomatic and economic relations with importing countries, contributing to broader trade diversification efforts. Exporting bioethanol also aligns with India's commitments to climate change mitigation and sustainable development goals, showcasing the country's leadership in promoting renewable energy solutions on the global stage [65].

To fully capitalize on the export potential of bioethanol, India needs to invest in infrastructure development, including transportation and storage facilities, to support efficient supply chain logistics. Moreover, continued investments in research and development to improve production efficiency, develop advanced technologies, and explore new feedstocks will enhance India's competitiveness in the global bioethanol market [28].

Overall, India stands poised to capitalize on its strengths in agriculture and biofuel production to become a significant bioethanol exporter, contributing to global energy security, environmental sustainability, and economic growth. With the right policies, investments, and strategic partnerships, India can position itself as a key player in the international biofuel arena.

### 4.4 Threats

#### 4.4.1 Climate vulnerability

Potatoes are indeed susceptible to climatic changes, making them vulnerable to fluctuations in temperature, precipitation, and extreme weather events. As a cool-season crop, potatoes have specific temperature and moisture requirements for optimal growth and development. Changes in climate patterns, such as rising temperatures, altered precipitation patterns, and increased frequency of extreme weather events like droughts or floods, can significantly impact potato cultivation [66].

High temperatures during the growing season can lead to heat stress, affecting potato plant physiology and reducing tuber formation and quality. Conversely, prolonged periods of cold or frost can damage potato plants, resulting in reduced yields and poor tuber quality. Changes in precipitation patterns, including shifts in rainfall timing and intensity, can also affect soil moisture levels, potentially leading to water stress or waterlogging, both of which can negatively impact potato growth and yield [67].

Furthermore, extreme weather events such as droughts, floods, or storms can cause physical damage to potato crops, disrupt planting and harvesting schedules, and increase the risk of pest and disease outbreaks, further compromising yield and quality. Additionally, climate change can alter the distribution and prevalence of pests and diseases that affect potatoes, posing additional challenges to potato cultivation [68].

Overall, the susceptibility of potatoes to climatic changes underscores the importance of climate-resilient agricultural practices and adaptation strategies to mitigate the impacts of climate variability and ensure the stability and sustainability of potato production systems. This may include the adoption of climate-smart agricultural practices, such as improved irrigation management, crop diversification, use of drought-tolerant potato varieties, and implementation of soil conservation measures to enhance resilience to climate-related stresses. Additionally, investments in research and development to develop climate-resilient potato varieties and crop management practices can play a crucial role in mitigating the adverse effects of climate change on potato cultivation and safeguarding food security and livelihoods for potato farmers.

#### 4.4.2 Global market fluctuations

Volatility in the global energy market can indeed have a significant impact on the profitability of bioethanol production. Bioethanol, as a renewable fuel, is closely linked to the broader energy market dynamics, particularly fluctuations in oil prices and government policies related to energy subsidies and mandates. When global oil prices are high, bioethanol may become more competitive as an alternative fuel, leading to increased demand and potentially higher profitability for bioethanol producers. Conversely, during periods of low oil prices, bioethanol may face greater competition from conventional fossil fuels, potentially reducing demand and profitability [53].

Moreover, government policies and regulations play a crucial role in shaping the market for bioethanol. Changes in biofuel blending mandates, tax incentives, tariffs, and trade agreements can influence the demand for bioethanol and its competitiveness relative to traditional fuels. For example, reductions in biofuel blending mandates or the removal of subsidies for bioethanol production can negatively impact the profitability of bioethanol producers, particularly in markets where biofuel consumption is heavily reliant on government support [69].

Additionally, global market fluctuations can affect the prices of key feedstocks used in bioethanol production, such as corn, sugarcane, and other agricultural commodities. Fluctuations in feedstock prices can directly impact production costs and, consequently, the profitability of bioethanol production. For instance, if the prices of feedstocks rise due to supply shortages or increased demand from other sectors, bioethanol producers may face higher production costs, potentially reducing profitability unless offset by corresponding increases in ethanol prices [38].

Furthermore, volatility in the global energy market poses both challenges and opportunities for the profitability of bioethanol production. Bioethanol producers must carefully monitor and adapt to changes in global energy prices, government policies, and feedstock markets to navigate market fluctuations effectively and maintain profitability in the dynamic and interconnected global energy landscape. Additionally, diversification of feedstock sources, investment in technology innovation, and strategic partnerships can help mitigate the impact of market volatility and enhance the resilience of bioethanol production operations.

#### 4.4.3 Pest and disease outbreak

The occurrence of widespread pest and disease outbreaks poses a significant threat to potato production, potentially leading to substantial yield losses and quality degradation. Potatoes are susceptible to various pests and diseases, including fungal, bacterial, and viral pathogens, as well as insect pests like aphids, potato beetles, and nematodes. Pest and disease outbreaks can occur due to a combination of factors, including environmental conditions, crop management practices, and the presence of susceptible potato varieties [1].

Fungal diseases such as late blight (caused by *Phytophthora infestans*) and early blight (caused by *Alternaria solani*) are among the most devastating diseases affecting potatoes worldwide. These diseases can spread rapidly under favorable environmental conditions, leading to foliage damage, tuber rot, and significant yield losses if left unmanaged. Bacterial diseases such as bacterial wilt (caused by *Ralstonia solanacearum*) and bacterial soft rot (caused by various bacteria in the genus *Pectobacterium* and *Dickeya*) can also cause severe damage to potato crops, affecting both plant health and tuber quality [70,71].

Moreover, viral diseases such as potato virus Y (PVY), potato leafroll virus (PLRV), and potato virus X (PVX) can lead to stunted growth, leaf yellowing, and reduced tuber yield and quality. Insect pests like the Colorado potato beetle (*Leptinotarsa decemlineata*) and aphids can transmit viral diseases and cause direct damage to potato foliage and tubers, further exacerbating yield losses [72,73].

The impact of pest and disease outbreaks on potato production can be mitigated through integrated pest management (IPM) strategies, including cultural practices, crop rotation, use of disease-resistant potato varieties, timely application of pesticides, and monitoring for early signs of pest and disease infestation. Additionally, research and development efforts focused on breeding disease-resistant potato varieties and developing environmentally friendly pest control methods can help enhance the resilience of potato crops to pest and disease pressure [66].

Overall, proactive management of pest and disease outbreaks is essential to safeguarding potato production and ensuring food security. Collaboration between farmers, researchers, extension services, and policymakers is crucial for implementing effective pest and disease management strategies and minimizing the economic and environmental impacts of pest and disease outbreaks on potato production.

#### 4.4.4 Policy changes

Government policies and mandates play a crucial role in shaping the landscape of the potato industry, particularly in the context of bioethanol production. Changes in government policies, regulations, and mandates can significantly impact the dynamics of the industry, posing both challenges and opportunities for stakeholders involved in potato-based bioethanol production [74].

For instance, policy changes related to biofuel blending mandates, tax incentives, tariffs, and trade agreements can directly affect the demand for bioethanol and its competitiveness relative to traditional fossil fuels. Reductions in biofuel blending mandates or the removal of subsidies for bioethanol production can adversely impact the profitability of bioethanol producers, particularly in markets where biofuel consumption is heavily reliant on government support. Conversely, supportive policies that incentivize bioethanol production, such as tax credits, subsidies, and renewable fuel standards, can stimulate investment in the industry and drive growth [75].

Moreover, broader agricultural policies, including those related to land use, water management, and agricultural subsidies, can indirectly influence the potato industry and its viability as a feedstock for bioethanol production. Changes in agricultural policies that affect potato cultivation practices, input costs, or market access can have ripple effects on the entire value chain, from potato growers to bioethanol producers [76].

On the other hand, regulatory frameworks related to environmental sustainability, food safety, and biotechnology can also impact the potato industry. Compliance with environmental regulations, such as restrictions on pesticide use or emissions standards for bioethanol production facilities, can add compliance costs and operational challenges for industry players. Similarly, regulations governing the approval and commercialization of genetically modified potato varieties with enhanced traits for bioethanol production can influence the adoption of innovative technologies in the industry [51,63].

In conclusion, while government policies and mandates can provide opportunities for growth and development in the potato-based bioethanol industry, they can also pose challenges if not aligned with the interests of industry stakeholders. Proactive engagement with policymakers, advocacy efforts, and collaboration between government and industry representatives are essential for shaping favorable policy environments that support the sustainable growth of the potato industry and its role in bioethanol production.

## 5 Conclusion and future thrust area

SWOT analysis of bioethanol production from potatoes in India highlights several strengths, weaknesses, opportunities, and threats has been presented. India's strengths lie in its abundant potato cultivation, which presents a readily available and potentially sustainable feedstock for ethanol production. Additionally, the country has a growing demand for renewable fuels and an established agricultural sector that can support bioethanol production. However, weaknesses such as high initial capital investment requirements, technological limitations, and infrastructure constraints pose challenges to the widespread adoption of potato-based ethanol production.

Despite these challenges, there are significant opportunities for India to capitalize on bioethanol production from potatoes. These include the potential to mitigate waste generation, enhance energy security, and tap into the growing global demand for renewable fuels. Moreover, technological advancements, research and development initiatives, and supportive government policies can further bolster the bioethanol industry in India.

However, it's crucial to acknowledge the threats to bioethanol production from potatoes in India, including competition from other feedstocks, market fluctuations, regulatory uncertainties, and environmental concerns related to land use and water consumption.

Looking ahead, future thrust areas for bioethanol production from potatoes in India should focus on addressing key challenges and leveraging opportunities. This includes continued investments in R&D to improve conversion efficiency, develop sustainable production methods, and explore innovative technologies for ethanol extraction from potatoes. Additionally, strategic partnerships between government agencies, research institutions, and industry stakeholders can facilitate knowledge exchange, technology transfer, and policy support to promote the growth of the bioethanol sector.

Finally, there is a need for targeted initiatives to enhance infrastructure development, strengthen supply chain logistics, and create awareness about the benefits of bioethanol as a renewable fuel. By addressing these key areas, India can unlock the full potential of bioethanol production from potatoes, contributing to sustainable development, energy independence, and environmental stewardship in the years to come.

## CRediT authorship contribution statement

**Dharmendra Kumar:** Writing – review & editing, Writing – original draft, Conceptualization. **Som Dutt:** Writing – review & editing, Conceptualization. **Arvind Kumar Jaiswal:** Writing – review & editing. **Bandana Kaundal:** Writing – review & editing, Writing – original draft. **Dinesh Kumar:** Writing – review & editing, Supervision. **Brajesh Singh:** Writing – review & editing, Supervision.

## Ethical considerations

Ethical approval is not applicable for this study.

## Participant consent

Participant consent is not applicable.

## Publication consent

Publication consent is not applicable.

## Data availability statement

Data included in article/supp. material is referenced in the article.

## Funding

This research received financial support from grants provided by the ICAR-Central Potato Research Institute, Shimla, India.

## Declaration of competing interest

The authors declare that they have no financial or personal relationships with any individuals or organizations that could inappropriately influence or bias the work presented in this manuscript. There are no potential conflicts of interest related to employment, consultancies, stock ownership, honoraria, paid expert testimony, patent applications or registrations, or any sources of grants or other funding. This statement is provided to ensure transparency and uphold the integrity of our research findings.
